# Adoptive NK cell therapy: a potential revolutionary approach in longevity therapeutics

**DOI:** 10.1186/s12979-024-00451-2

**Published:** 2024-06-26

**Authors:** Xuewen Deng, Hiroshi Terunuma

**Affiliations:** 1 Biotherapy Institute of Japan, Inc. 2-4-8 Edagawa, Koto-Ku, Tokyo, 135-0051 Japan; 2 N2 Clinic Yotsuya, 5F 2-6 Samon-Cho, Shinjuku-Ku, Tokyo, 160-0017 Japan

**Keywords:** NK cell therapy, Immunosenescence, Senescent cells (SNCs), Aging-associated diseases (AADs)

## Abstract

The aging process intricately involves immune system dynamics, with a crucial role in managing senescent cells (SNCs) and their senescence-associated secretory phenotypes (SASPs). Unfortunately, immunosenescence, a progressively dysregulated immunity with age, hampers effective SNC elimination, leading to accumulation, coupled with the release of SASPs, which, in turn, inhibits immunity and heightened susceptibility to aging-associated diseases (AADs). Natural killer (NK) cells, integral to the innate immune system, play a pivotal role in addressing SNCs swiftly. These cells also coordinate with other components of both innate and adaptive immunity to surveil and eliminate these cells. Accordingly, preserving NK cell function during aging is crucial for evading AADs and promoting healthy aging. Alternatively, NK-cell-based therapies present promising avenues for addressing the challenges associated with aging. Notable, recent studies in adoptive NK cell therapy have shown promise in rejuvenating immunosenescence, eliminating SNCs, and alleviating SASPs. This progress provides the proof-concept of adoptive NK cell therapy for senotherapy and holds promise as an emerging revolution in longevity therapeutics.

## Introduction

As the global population ages, the prevalence of associated diseases becomes increasingly apparent [1]. The pursuit of healthy aging, characterized by heightened resistance to lethal diseases, is the cornerstone of preventive medicine [2]. The aging process is a complex process involving cellular senescence and inflammation [3, 4], with the immune system playing a pivotal role in managing these aspects [3–5]. Timely clearance of senescent cells (SNCs) is central to maintaining tissue and organismal homeostasis [6, 7]. Unfortunately, immunosenescence, a progressively dysregulated immune state with age [8], fails to eliminate SNCs, leading to their accumulation. This often coincides with the release of senescence-associated secretory phenotypes (SASPs), inhibiting immunity and increasing vulnerability to aging-associated diseases (AADs) [3, 9, 10]. Consequently, targeting immunosenescence and SNCs emerges as a crucial therapeutic strategy to preserve and extend healthy aging [8, 11, 12]. While adaptive immunity has traditionally taken center stage in immunogerontological studies [11], growing evidence underscores the substantial impact of innate immunity in AADs [3–5]. Natural killer (NK) cells, integral to the innate immune system, uniquely identify and eliminate aberrant cells such as tumor and virus-infected cells [12–16]. Moreover, NK cells promptly address SNCs [4, 6, 7], and coordinate with other immune components through cytokine and chemokine production to surveil and eliminate cancer cells [17, 18]. Although whether the same occurs against SNCs remains to be determined. Evidence from healthy elderly individuals, especially those exhibiting physical fitness, independence in daily activities, or adequate cognitive function, the number and function of NK cells are highly preserved [19–22]. Conversely, diminished NK cell activity in elderly individuals is associated with disorders such as atherosclerosis [23] and an elevated risk of mortality [24, 25]. Accordingly, preserving NK cell function during aging is deemed crucial for healthy aging and longevity [4, 9]. Alternatively, NK-cell-based therapies, notably adoptive NK cell therapy, aligning with their established role in cancer and viral infection treatments [13, 16], show promise in rejuvenating immunosenescence, eliminating SNCs and alleviating SASPs, that lead to AADs [26–28]. This short review will delve into this issue to exploring the potential of adoptive NK cell therapy in fostering healthy aging and longevity.

### Cellular senescence and aging-associated diseases

Cellular senescence, marked by irreversible cell growth arrest [29], serves crucial roles in development and tissue repair [6, 7], preventing abnormal cell proliferation and suppressing tumor growth [30] to maintain tissue homeostasis. However, with aging, SNCs progressively accumulate, often releasing SASPs [31]. This excessive presence of SNCs and SASPs disrupts tissue function, impacting neighboring cells and standing as a key contributor to AADs [10, 32, 33], ultimately curtailing the healthy lifespan [34]. Distinct populations of SNCs drive specific AADs, encompassing cancer [35], cardiovascular diseases [36], neurodegenerative diseases [37], and osteoarthritis [38]. Senolytic drugs, proven to eliminate SNCs in animal models and mitigate SASPs, exhibit promise in delaying or alleviating age-associated pathologies while extending lifespan [39]. Nevertheless, translating senolytic drugs to human use faces challenges due to the heterogeneity of SNCs and potential toxicity risks [40]. Therefore, exploring alternative methods that safely eliminate SNCs in humans is imperative.

### NK cells: guardians against senescent cells

NK cells, emerging from the bone marrow, play a crucial role in identifying and eradicating aberrant cells, including SNCs [4, 13–16]. Beyond their direct elimination prowess, NK cells contribute to immune responses by secreting cytokines and chemokines, collaborating with other components of innate and adaptive immunity [17, 18]. Constituting 10–20% of the circulating lymphocyte pool, NK cells exhibit distinct subsets based on CD56 expression density. Roughly 90% of peripheral blood NK cells are CD56^dim^, known for heightened cytotoxicity but lower cytokine and chemokine production upon activation. In contrast, the 10% CD56^bright^ NK cells excel in proliferation and produce a diverse array of cytokines and chemokines, albeit with minimal cytotoxicity [19]. The delicate equilibrium of NK cell function hinges on the interplay of activating and inhibiting signals through receptor interactions with ligands on target cells [41]. A plethora of NK cell receptors, ligands, and associated functions have been identified [42]. When activating signals surpass inhibitory ones beyond a critical threshold, NK cells spring into action, eliminating aberrant cells and secreting cytokines and chemokines that synchronize with other immune components. Notably, MHC class I chain-related (MIC) A/B and poliovirus receptor CD155, expressed on SNCs, act as activating receptor ligands (NKG2D and DNAM-1, respectively) [43]. Studies in vitro and in animal models have shown that NK cells actively eliminating SNCs [44, 45]. Thus, NK cells wield the ability to immune surveillance SNCs, instigating a response for their elimination [4, 5]. The orchestrated clearance of SNCs by NK cells is pivotal for upholding tissue homeostasis [4, 6, 7].

### NK cell immunosenescence and its impact on aging-associated diseases

**Fig. 1:**
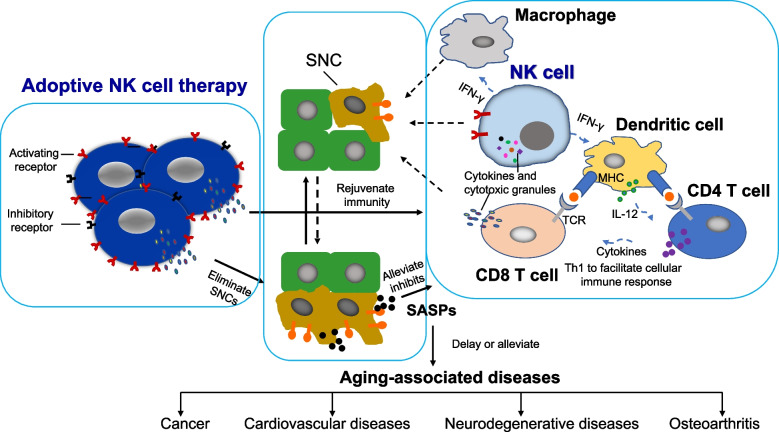
The schematic illustration outlines the potential of adoptive NK cell therapy in longevity therapeutics. Adoptive NK cell therapy directly targets and remove senescent cells (SNCs), resulting in the alleviation of senescence-associated secretory phenotypes (SASPs) and the rejuvenate and/or restore immunity. This intervention breaks the vicious cycle, ultimately reducing the burden of aging-associated diseases (AADs) (solid lines). Abbreviations: AADs, aging-associated diseases; NK cell, natural killer cell; SASPs, senescence-associated secretory phenotypes; SNCs, senescent cells

The immune system is pivotal in handling both foreign organisms and aberrant cells [8, 13–16]. Numerous studies underscore the involvement of immune cells—macrophages, NK cells, and cytotoxic T cells—in the ongoing surveillance of SNCs [4, 12]. Immunosenescence, a process characterized by declining immunity with age, is a hallmark of aging that affects both innate and adaptive immunity. It involves alterations in immune cells numbers, phenotypic and functional changes, and extends beyond defects in the clearance of SNCs, contributing to another hallmark of aging-inflammation [15, 46]. This decline results in the evasion of SNCs from immune detection, leading to their persistent accumulation and the release of SASPs [3]. This cumulative immunosenescence significantly contributes to the heightened occurrence of chronic conditions in older individuals, collectively referred to as AADs, encompassing cancer, cardiovascular diseases, neurodegenerative diseases, and osteoarthritis [35–38] (Fig. 1). The aging process brings about distinct changes in human NK cells. This includes a gradual decrease in the CD56^bright^/CD56^dim^ subset ratio, phenotypic alterations (e.g., reduced expression of activating receptors like NKp30), an expansion of CD56^dim^ CD57^+^ NK cells, and a decline in cytokine secretion and cytotoxicity against target cells [15, 20, 47–50]. Although the age-associated impairment of NK cells is well-documented in the context of tumor cells, it necessitates further elucidation regarding their efficacy in eliminating SNCs. Nonetheless, the immunosenescence of human NK cells emerges as a significant contributor to AADs [9, 12, 51], given their pivotal role in surveilling SNCs, the accumulation of which is a primary driver of SASPs with aging [10, 32, 33] (Fig. 1). NK cells, as innate immune responders, directly eliminate SNCs through granule exocytosis, with death-receptor pathways playing a limited role [52]. Furthermore, NK cells collaborate with other immune cells, such as macrophages and cytotoxic T cells, forming an interconnected network for comprehensive immunosurveillance against SNCs [17, 18] (Fig. 1). Impaired immunosurveillance accelerates the accumulation of SNCs and SASPs [52]. In light of this, the development of intervention strategies to rejuvenate or restore immunity, including NK cell function, holds promise for preventing and treating AADs. This approach envisions individuals aging without the burden of diseases [11]. Nonetheless, human studies emphasize that extrinsic factors, particularly lifestyle choices, significantly impact the number and function of NK cells [15]. Hence, promoting a healthy lifestyle becomes imperative, offering a favorable avenue to prevent NK cell immunosenescence and fostering successful aging [15].

### Adoptive NK cell therapy: a cutting-edge approach for aging and aging-associated diseases

Adoptive NK cell therapy entails introducing ex vivo activated NK cells directly into a patient. As meticulously reviewed by Myers et al., there’s ongoing exploration of various sources for therapeutic NK cells, potentially customizable to target SNCs [53]. Given their broad cytotoxicity and collaborative interactions with innate and adaptive immunity, NK cells emerge as promising candidates for senotherapy. Autologous NK cell therapy, involving the expansion of a patient’s own NK cells ex vivo, offers practical advantages in terms of ease of procurement and sidesteps challenges associated with HLA mismatch. This approach has undergone safety and tolerance assessments in cancer patients [54, 55]. Our research group has pioneered a feeder-cell-free method for substantial NK cell expansion, resulting in heightened expression of activating receptors, increased cytotoxicity, and elevated cytokine production compared to resting NK cells for clinical studies [54, 56]. Notably, these expanded NK cells demonstrated superior cytotoxicity against senescent fibroblasts in vitro [27]. Recent human studies underscore the effectiveness of adoptive autologous NK cell therapy in eliminating SNCs from peripheral blood mononuclear cells (PBMCs) and CD3^+^ T cells, as evidenced by markers p16 and β-galactosidase for up to 90 days post-infusion [27, 28]. This led to reduced specific T cell subsets, along with a decrease in well-defined inflammatory cytokines in both human [26] and mice [28]. This suggests a rejuvenated immunity, a reduced inflammatory burden [26, 28], and diminished senescence in various tissues of aged mice post-NK cell infusion, including the liver, kidney, lung, fat, and eye [28]. Moreover, documented cases reveal that two NK cell infusions alleviated human immunosenescence for more than a year [27]. Importantly, SNCs can activate NK cells, evidenced by increased expression levels of CD69 and perforin [28]. In essence, adoptive NK cell therapy holds the potential to rejuvenate and restore immunity by eliminating SNCs from PBMCs and tissues, thereby alleviating immunosenescence and SASPs (Fig. 1). This groundbreaking approach has transformative potential to mitigate the detrimental features of AADs, marking an innovative frontier in ultimate preventative medicine. Nevertheless, these results are not sufficiently powered to draw conclusion on longevity at this stage. Moreover, considering the therapeutic efficiency, establishing the preferred optional culture condition is crucial to ensure the expansion is non-senescent NK cells for improving NK cell functions. Furthermore, it would be necessary to verify whether aging impacts the expansion of NK cells.

## Advantages of adoptive NK cell therapy compared with existing therapeutic modalities

Recent medical innovations have led to development of various methods and strategies to rejuvenate the immune system, such as nutrition, exercise, hormonal products, and other supplements. It must be stated that no well-established method beneficially impacts on the very complex nature of immunosenescence yet [11, 25]. There is also growing interest in the development of small-molecule drugs that target poorly defined molecular on SNCs and must be administered repeatedly overtime, producing substantial side effects [40]. Unlike small molecules, adoptive NK cell therapy has undergone safety and tolerance assessments and has the potential to persist and mediate the potent effects after single administration [26, 27]. Additionally, NK cell can migrate to SNCs in normal tissues by the local inflammatory environment produced by SNCs and eliminate them [28]. This in contrast to the immunosuppressive microenvironment created by tumor cells, which may impede the therapeutic effects. Consequently, adoptive NK cell therapy may have broad therapeutic potential mitigate the detrimental features of AADs.

### Mechanistic insights and biomarkers of adoptive NK cell therapy for serotherapy

NK cells communicate with other immune components and promptly address SNCs [4, 6, 7, 44]. Chelyapov et al., described a studying in vitro where activated NK cells attack SNCs on highly cooperated level [27]. They assessed the senescent markers, p16 and β-galactosidase, in PBMCs before and after adoptive NK cell therapy from 5 healthy individuals, supporting the removal of immunosenescent cells in human [27]. Similarly, Tang et al., uncovered that senescence and exhaustion T cells were eliminated, and the secretions of SASP factors were decreased after adoptive NK cell therapy [26].

Urokinase plasminogen activator receptor (uPAR), upregulated on SNCs across different cell types, was paralleled with β-galactosidase-positive cells. Plasma levels of soluble uPAR positively correlate with the pace of aging in humans. uPAR chimeric antigen receptor (CAR) T cells can eliminate SNCs and improve aging-associated metabolic dysfunction [57]. Therefore, uPAR may serve as a suitable candidate biomarker of therapeutic efficacy.

## Conclusions and future directions

Addressing aging and AADs through the immune elimination of SNCs and the management of age-related inflammation emerges as a promising strategy [4, 5, 12]. The evolution of NK cell research from its established roles in anti-tumor and viral immunity to the clearance of SNCs marks a significant advancement. Although in its early stages, initial studies on adoptive NK cell therapy showcase promising outcomes, including the mitigation of immunosenescence and SASPs, eliminating SNCs [26–28] (Fig. 1). While these preliminary findings are encouraging, further exploration is essential. Understanding the intricate mechanisms, clarifying the in vivo mode of action, determining the optimal timing for intervention in the aging process, and establishing the most effective therapeutic NK cell dosage are crucial aspects for deeper insights. Robust clinical trials involving larger cohorts are imperative to confirm the therapeutic role in addressing AADs and extending lifespan. Moreover, investigating alternative sources of therapeutic NK cells, such as allogenic NK cells, umbilical cord NK cells, stem cell-derived NK cells, and CAR-NK cells, holds substantial promise. Unraveling the complexities of preventing, delaying, or reversing NK cell immunosenescence will propel the revolution in longevity therapeutics, marking a paradigm shift in ultimate preventative medicine.

## Data Availability

No datasets were generated or analysed during the current study.

## Abbreviations

AADs: Aging-associated diseases
CAR: Chimeric antigen receptor
NK cells: Natural killer cells
PBMCs: Peripheral blood mononuclear cells
SASPs: Senescence-associated secretory phenotypes
SNCs: Senescent cells
uPAR: Urokinase plasminogen activator receptor
